# Excess length of stay and economic consequences of adverse events in Dutch hospital patients

**DOI:** 10.1186/s12913-015-1205-5

**Published:** 2015-12-01

**Authors:** Janneke Hoogervorst-Schilp, Maaike Langelaan, Peter Spreeuwenberg, Martine C. de Bruijne, Cordula Wagner

**Affiliations:** NIVEL, Netherlands Institute for Health Services Research, PO Box 1568, 3500 BN Utrecht, The Netherlands; Department of Public and Occupational Health & EMGO Institute for Health and Care Research, VU University Medical Centre, Van der Boechorststraat 7, 1081 BT Amsterdam, The Netherlands

**Keywords:** Adverse event, Excess length of stay, Costs, Hospital

## Abstract

**Background:**

To investigate the average and extrapolated excess length of stay and direct costs of adverse events (AEs) and preventable AEs in Dutch hospitals, and to evaluate patient characteristics associated with excess length of stay and costs.

**Methods:**

Data of a large retrospective patient record review study on AEs was used. A stratified sample of 20 Dutch hospitals was included. Excess length of stay and costs attributable to AEs and preventable AEs were calculated and extrapolated to a national estimate. The association between patient characteristics and excess length of stay (and costs thereof) attributable to AEs and preventable AEs was investigated through multilevel linear regression analyses.

**Results:**

A total of 2975 patient records were included in the analysis, of which 325 experienced one or more AEs. Hospital patients experiencing an AE stayed 5.11 (95 % CI 3.91–6.30) more days in hospital and cost €2600 (95 % CI €1968–€3232) more compared to those without an AE. There was no significant difference in days and costs between preventable and non-preventable AEs. Extrapolated to a national level, AEs cost more than €300 million, which was 1.3 % of the national hospital care budget. Patients with hospital-acquired infections had a statistically significant longer length of stay compared to the reference group (patients with AEs on the cardiovascular system).

**Conclusions:**

This study showed that AEs lead to substantial excess length of stay and increased costs. Special attention should be paid to patients with AEs due to an hospital-acquired infection.

## Background

An AE is defined as an unintended injury that results in temporary or permanent disability, death or prolonged hospital stay, and is caused by healthcare management rather than by the patient’s underlying disease process [1]. Between 2.9 and 16.6 % of hospital patients experienced an AE during their hospital admission [1–8]. Several studies showed that AEs are associated with longer hospital length of stay (LOS). Excess LOS attributable to AEs ranges from 6 to 9 days [9–14], leading to higher healthcare costs [15–25]. Earlier studies investigating excess LOS and associated costs of AEs were mostly limited to AEs caused by medication errors [15, 19, 23–25], or investigated only costs associated with specific diseases or treatment [17, 21, 22]. In the Netherlands, total direct medical costs of AEs in hospitals were estimated using patient records of 2004 [18]. The total annual direct medical costs were estimated at €355 million for all AEs. Since 2004, much has changed in the Dutch healthcare system and utilisation of hospital care. Diagnosis Related Groups (DRG’s) were introduced in hospital care in 2005 to improve transparency and quality, by defining products which can be negotiated between purchasers and providers of care. The total hospital care costs rose from €15.5 billion in 2004 to €23.9 billion in 2012, forcing hospitals to reduce healthcare costs. Since the major part of the direct medical costs in hospitals due to an AE consists of excess LOS [18], insight into the excess LOS and costs associated with AEs is essential for determining economic consequences of AEs and preventable AEs. This information may support hospitals in justifying their investments in patient safety interventions that will both improve patients’ health and reduce avoidable health system costs.

Besides the economic consequences of AEs, information should also be available about the patient’s characteristics contributing to a longer LOS and higher costs if an AE occurs. An earlier study showed that poorer health status during admission was associated with increased AEs, and also with increased costs and LOS [26]. The study of Hoonhout et al. showed which determinants influenced the costs attributable to AEs in 2004.

The aim of this study is to investigate the average and extrapolated excess LOS and direct costs of AEs and preventable AEs in Dutch hospitals. Furthermore, patient characteristics associated with excess LOS and costs due to AEs and preventable AEs will be evaluated.

## Methods

### Design and setting

For the present study, data of a large retrospective patient record review study was used. This study was performed in a random sample of 20 Dutch hospitals, stratified by hospital type and location (urban or rural). Eligible hospitals had at least 200 beds, an emergency department and an intensive care unit. A total of four university-, eight tertiary teaching- and eight general hospitals were included in the sample. Tertiary teaching hospitals in The Netherlands provide specialised care and train doctors. The complexity of care given is between that given in a university hospital and in a general hospital. In each participating hospital, 200 patient admissions between April 1st 2011 and March 31st 2012 were randomly selected A large subsample (50 % of the sample) of hospital patients who died in hospital during admission, was included. This made it possible to estimate the number of preventable deaths in the primary study as this is a relatively small patient group. These patients were sampled from all inpatient deaths The random sampling was performed by giving random numbers to eligible patients (with Excel) and to include number 1–100 of the deceased patients and number 1–100 of the alive patients. Patients admitted to the psychiatry department, obstetrics and children <1 year old were excluded. More details about the design and methods of the study have been described elsewhere [27].

### Patient record review

The nursing, medical, and – if available – outpatient records of the sample patient admissions were reviewed by 34 trained external nurses and 17 trained external medical specialists (eight internists, seven surgeons and two neurologists) in a two-stage review process. The method of determining AEs was comparable to those of other international studies [2, 3]. In the first stage a nurse screened the records by using 16 triggers indicating potential AEs. In the second phase, admissions positive for at least one trigger were further reviewed by a physician. Based on a standardised procedure, and preceded by a number of underlying questions to secure a systematic assessment, the presence and preventability of an AE was determined. An AE was defined by three criteria:1) an unintended (physical and/or mental) injury which results in; 2) prolonged hospital stay, temporary or permanent disability or death; and 3) is caused by healthcare management rather than the patient’s condition.

An AE was found to be preventable when the care given fell below the current level of expected performance for practitioners or systems. The cause and preventability of AEs were scored on a six-point Likert scale. A score of 4–6 indicated that the reviewer regarded the event as having a greater than 50 % chance of being caused by healthcare, or was preventable, and was considered as AE.

The AEs that occurred during the patient’s index hospital admission and were detected during either the index admission or subsequent admissions over the following 12-month period were counted. AEs related to patient admissions in the same hospital within the 12 months preceding the index admission, but which were not detected until the index admission were also counted. Consequently, patient records of the index hospital admission were reviewed, as were the patient records of patient admissions before and after the index admission.

Additional data on the total Dutch hospital population in 2011/2012 and administrative hospital information for the reviewed patient admissions was collected from the national hospital administration database (LMR) maintained by Dutch Hospital Data.

### Excess length of stay

Within the LMR, for each hospital admission, the expected LOS was computed. This calculation was based on characteristics of the patients and the national mean LOS that is associated with these characteristics [28]. The following characteristics of the patients were taken into account:

- Age, divided into four classes: 1–14, 15–44, 45–64, 65+ years
- The primary diagnosis that resulted in the admission, including about 1,000 diagnoses classified by the ICD9 in three digits
- Procedures, classified by the Dutch Classification System of Procedures. The procedures considered depend on the diagnosis of the patient.

Normally these calculations were only used for patients discharged alive, but for the purpose of this study the expected LOS was also calculated for the patients which died in hospital. The excess LOS during the index admission was calculated by subtracting the expected LOS from the actual observed LOS, using the LMR data. If expected LOS in was missing within the LMR, the excess LOS was imputed by estimations of extra hospital days due to an AE indicated by the reviewer during the second stage of the study. The estimates of excess LOS varied from no extra bed days to all bed days of the index admission. For re-admissions attributable to AEs during the index admission, we imputed the national average hospital stay in 2012 (5.3 days).

### Costs of excess LOS

To estimate direct costs of excess LOS, the calculated excess LOS during index admission and re-admission was multiplied by unit costs. The costs of one extra hospital admission day was valued by the Dutch guideline prices of 2009 [29]. Only guideline prices for university and general hospitals were available. For tertiary medical teaching hospitals, guideline prices for general hospitals were used. The unit costs include costs of a standard hospital day, medical and nursing staff, medication, material, equipment, housing and overhead. After correction with price indices for 2012, the unit costs per day were €461.83 for general- and tertiary medical teaching hospitals and €610.46 for university hospitals. The unit costs per day were €2317.63 for intensive care units (ICUs).

### Potential determinants

Several patient characteristics were investigated as potential determinant for excess LOS and costs due to AEs and preventable AEs. Age (in years), sex, urgent admission (yes/no), and specialty (surgery versus non-surgery) were collected within the patient record review. ICD9 diagnostic information was gathered from the LMR data and used to define diagnostic groups. The following diagnostic groups were defined: neoplasm, endocrine, nutritional and metabolic diseases, cardiovascular diseases, respiratory system, digestive system, genitourinary system, symptoms, signs and ill-defined conditions, and injury and poisoning. All other diagnostic codes were defined as ‘all other codes’.

Besides patient characteristics, type of AE was also investigated as potential determinant for excess LOS and costs due to AEs and preventable AEs.

### Statistical analysis

The mean excess LOS and costs attributable to AEs and preventable AEs with 95 % confidence intervals (CIs) were estimated using multilevel linear regression analyses. A two-level multilevel structure was used, whereby the observations were clustered within hospitals. The national estimate of excess LOS and costs attributable to AEs in 2011/2012 was calculated by multiplying total amount of Dutch hospital admissions in the period April 1st 2011 and March 31st 2012 (*n* = 1.678.283) with AE rate (7.1 %) [30] and by subsequently multiplying this with the extra costs of an AE. The calculation was repeated for costs of preventable AEs (1.6 % preventable AE rate).

Multilevel linear regression analyses were performed to investigate the association between patient characteristics and excess LOS attributable (and the costs thereof) to AEs and preventable AEs. The dependent variable in the model was either the excess LOS or the costs of excess LOS. The independent variable was either AE or preventable AE. The determinants were added as covariates to the models. The variables included in the model were centred to reference values for all Dutch hospital admissions in 2011/2012. The variables ‘type of AE’ and ‘diagnostic groups’ were centred to the mean of these variables in the total sample.

The amount of variation in excess LOS and costs of AEs and preventable AEs caused by hospital level was indicated by the Intraclass Correlation Coefficient (ICC) [31]. The ICC is the ratio of between-group variance and total variance, whereby a higher ICC represents a larger variance between hospitals in AE rates and a smaller variance within hospitals.

An additional analysis was performed to investigate the costs of excess LOS including ICU days, as the costs of ICU days are much higher compared to the costs of a normal hospital day. ICU costs were not included in the main analysis, because we had no information about the exact number of ICU days caused by the AE. For patients admitted to the ICU during their index admission, the number of days in ICU or intermediate care, reported in the first stage of the review process, was multiplied by unit costs of an ICU day. The remaining days of the excess LOS were multiplied by unit costs per day for the specified hospital type.

In all multilevel analyses, corrections were made for overrepresentation of patients admitted to a university hospital and for overrepresentation of patients who died in hospital by adding these covariates to the model. Descriptive analyses were performed using Stata 13.0. Multilevel analyses were performed using MLwiN version 2.24 (University of Bristol, 2011). For all analyses, *p*-values ≤ .05 were considered statistically significant.

## Results

During the first stage, a total of 4048 patient records were reviewed by nurses. In the present study, patient records with missing data for both expected LOS in the LMR data and estimations of excess LOS by the reviewer during the second stage of the study (*n* = 915) and with missing data on any of the potential determinants for excess LOS (*n* = 22) were excluded, resulting in a sample of 3111 patient records. LOS was imputed for 142 patient records (4.5 %) by the estimations indicated by the reviewer. During the second stage, physicians identified one or more AEs in 348 of these 3111 reviewed patient admissions. For patients with multiple AEs, only records with the first reported AEs (*N* = 325) were included in the analysis, resulting in a total sample of 2975 patient records. AEs were found to be preventable in 89 of the 325 patient records. These numbers are unweighted.

### Excess LOS and costs of (preventable) AEs

Table 1 shows mean excess LOS and costs attributable to AEs following from the multilevel linear regression analyses. The LOS of patients experiencing an AE was 5.11 days (95 % CI 3.91–6.30) longer compared to patients without an AE. The mean extra costs of LOS were €2600 (95 % CI 1968–3232) higher for patients experiencing an AE compared to those without an AE. There was no statistically significant difference (mean difference 2.33 days; 95 % CI −1.19–5.85) in LOS between preventable and non-preventable AEs. There was no statistically significant difference (€1021; 95 % CI −775–2817) in costs between preventable AE and non-preventable AEs.

**Table 1:** Mean excess LOS and costs of AEs and preventable AEs in the Netherlands in 2011/2012^a^

	*N*	Mean excess LOS in days (95 % CI)	Mean costs of excess LOS in € (95 % CI)	Sensitivity analysis mean costs of excess LOS in € (95 % CI)^c^
AE (*N* = 2975)				
No	2650	2.43 (1.51–3.35)	1085 (695–1475)	1084 (354–1814)
Yes	325	7.53 (6.12–8.94)***	3685 (2992–4378)***	7122 (5577–8667)***
Preventable AE (*N* = 325)^b^				
No	236	7.94 (5.28–10.60)	3906 (2580–5233)	4982 (1479–8484)
Yes	89	10.26 (6.52–14.00)	4927 (3034–6821)	7446 (2449–12443)

*AE* adverse event, *LOS* length of stay; ***P < 0.001

^a^In all multilevel linear regression analyses mean excess LOS were corrected for overrepresentation of patients admitted to a university hospital and of deceased patients

^b^Number of patients experiencing an AE

^c^Costs of ICU days were included in the sensitivity analysis

Extrapolating differences in costs due to an AE resulted in an estimate of €309,812,233 extra costs attributable to AEs in the Netherlands in 2011/2012. This was 1.3 % of the national hospital care budget (€23.9 million) in 2012. Per 100,000 patient admissions, the estimated extra costs attributable to AEs were €18,460,071.

During the index admission, patients experiencing an AE were more often admitted to intensive care or intermediate care (45.5 %) compared to those without an AE (21.0 %). The additional multilevel analysis showed a larger difference in costs (€6038; 95 % CI 4543–7533) between those experiencing AEs compared to those without AEs when the ICU costs were included. There was no statistically significant difference (€2464; 95 % CI −2301–7230 ) in costs between preventable AEs and non-preventable AEs.

#### Determinants of excess LOS and costs

Table 2 shows the results of the multilevel linear regression analysis of excess LOS and costs due to AEs. Excess LOS differed statistically significantly between different types of AE. Patients with a hospital-acquired infection had a statistically significant longer excess LOS of 4.7 days and €2337 higher costs compared to those with AEs on the cardiovascular system. The costs of the excess LOS due to AEs were €537 lower for urgent admissions compared to those for planned admissions. No other statistically significant determinants were found.

**Table 2:** Multilevel regression analysis of the excess LOS and costs of AEs (*N* = 2975)

	AE				
	*N* (%)	Excess LOS		Costs	
		Days	SE	€	SE
AE (ref = no AE)	325	5.03***	0.62	2536***	192
Age		−0.003	0.01	−3	6
Sex (ref = men)	1555 (52)	0.26	0.38	163	200
Urgent admission (reference = no)	2073 (70)	−0.70	0.49	−537*	262
Surgery specialty (ref = non-surgery)	976 (33)	−0.67	0.48	−385	253
Type of AE (ref = cardiovascular system)					
Respiratory system	34	0.18	2.55	225	1355
Renal system	29	−0.14	2.64	−78	1401
Haematological system	20	2.27	2.89	1494	1536
Gastrointestinal event	30	−1.15	2.58	−706	1370
Neurological system	18	−3.32	2.94	−1653	1563
Hospital-acquired infection	79	4.70*	2.08	2337*	1104
Surgical or obstetric event	76	2.04	2.09	956	1112
Other type of AE	23	4.71	2.74	2415	1457
Diagnostic groups (ref = infection diseases)					
Neoplasm	515 (17)	0.71	1.12	317	594
Endocrine, nutritional and metabolic diseases	57 (2)	−0.18	1.70	−205	902
Cardiovascular diseases	759 (26)	−1.06	1.09	−595	577
Respiratory system	309 (10)	0.10	1.17	56	622
Digestive system	276 (9)	1.29	1.20	781	637
Genitourinary system	147 (5)	−0.45	1.34	−311	710
Symptoms, signs and ill-defined conditions	152 (5)	0.29	1.31	156	698
Injury and poisoning	266 (9)	0.14	1.22	141	648
All other codes	494 (17)	−0.09	1.17	−112	623
ICC^a^ hospital level (%)		5.0 %		0.01 %	

*AE* adverse event, *LOS* length of stay, *SE* Standard Error, *β* difference;*P < 0.05; ***P < 0.001

^a^Intraclass correlation (ICC) is the ratio of the between-group variance and the total variance

The multilevel analysis of excess LOS and costs due to preventable AEs showed no statistically significant determinants for excess LOS due to preventable AEs compared to non-preventable AEs (table not shown).

#### Variation in excess LOS and costs

The variation in excess LOS was not caused by hospital level as the ICC was 0.05 for AEs and 0.00 for preventable AEs. A comparable result was found for variation in costs, the ICC was 0.01 for AEs and 0.00 for preventable AEs. This means that at most only 5 % of the variance in excess LOS and costs was caused by differences in the investigated determinants between individual hospitals. The remaining variance is caused by differences in other characteristics such as patient characteristics, organisation characteristics, medical staff characteristics, technology characteristics, or environment.

## Discussion

The results of our study showed that patients experiencing an AE during hospitalisation stay five days longer in hospital and cost €2600 more compared to those not experiencing an AE. Extrapolation of our results showed that total costs of excess LOS due to AEs was more than €300 million in Dutch hospitals in 2011/2012. This was 1.3 % of the Dutch national hospital care budget in 2012. AEs concerning hospital-acquired infections contributed to longest excess LOS and highest costs. Urgently admitted patients had lower costs of excess LOS due to AEs compared to planned admissions. No statistically significant difference in days and costs was found between preventable and non-preventable AEs.

The mean excess LOS due to AEs was slightly lower compared to results of previous studies in hospital patients. Naessens et al. found a difference in excess LOS of 8.6 days between hospital patients with and without an AE [26]. Ehsani et al. found a difference of 7.0 days between patients with acute admission for cardiac disease experiencing AEs and those without AEs [16]. The costs of excess LOS investigated in these two studies could not be compared to our results, as they estimated total costs of patients from hospital costing systems. Other recent studies considering costs of LOS investigated only excess LOS due to adverse drug events, and could therefore not be compared to our results [15, 19, 24]. Compared to the results of 2004 [18], the excess LOS was almost half of the days (5.1 compared to 9.1 days). A reduction could have been expected, as there was a nationwide downward trend in mean hospital LOS in the last decade, from 7.3 days in 2004 to 5.3 days in 2012. Possibly, increased awareness and earlier recognition of consequences of AEs among healthcare providers contributed to more rapid action being taken.

Following the results of our study, patients with hospital-acquired infections had a statistically longer LOS and higher costs. This is in line with an observational study in hospital patients, showing that a hospital-acquired infection with Clostridium difficile increased the medium LOS by 6 days [32]. Implementation of possibilities for preventing these infections should be investigated, for example improving hand hygiene [33]. Urgently admitted patients had lower costs attributable to AEs compared to planned admissions. This might be the result of differences in the percentage of urgent admissions between hospital types: in the more expensive academic hospitals fewer patients were urgently admitted (59 %) compared to tertiary medical teaching hospitals (71 %) and general hospitals (75 %).

An important strength of this study is that complete patient admissions have been reviewed independently by qualified nurses and physicians in a structured manner to ascertain AEs. Another strength is the large sample of hospitals and stratification by hospital type and location, which enabled extrapolation of the results to a national level.

Some limitations of our study have to be mentioned. Despite the correction for the overrepresentation of deceased patients, the calculated excess LOS might be slightly overestimated. Deceased patients are probably more severe patients compared to the discharged patients. An additional analysis showed that the deceased patients had a statistically significant (*P* < 0.001) longer excess LOS of 1.67 days (95 % CI 0.88–2.45) compared to the discharged patients. The calculated costs of AEs in our study were presumably an underestimation of the total costs within hospitals caused by AEs, because only costs of excess LOS were included. Costs of extra medical procedures attributable to AEs could not be taken into account as the registration of these procedures was poor. This is a result of the system (Diagnosis-related group, DRG), which reimburses hospitals based on fixed prices for a combination of diagnosis and treatment, whereby procedures are not declared individually. Hereby, the results of our study could not be compared directly to other countries with different health care and cost systems. The method used for calculating excess LOS (observed LOS minus expected LOS) may also have contributed to an underestimation of the excess LOS [28]. However, this was the most accurate available method to approach excess LOS due to an AE. From a societal perspective, outpatient healthcare, productivity loss and loss of income could also be important, as are the potential costs for medical claims after an AE occurred. However, these outcomes could not be taken into account in our study. Despite these limitations in calculating excess LOS, our study showed that AEs leads to substantial excess LOS and costs.

## Conclusions

This study showed that AEs leads to substantial excess LOS and increased costs. No statistically significant difference was found in excess LOS and costs between preventable and non-preventable AEs. Extrapolating the results showed that the total costs of excess LOS due to AEs in Dutch hospitals was more than €300 million in 2011/2012. These costs were approximately 1.3 % of the national hospital care costs. Since patients with AEs due to an hospital-acquired infection had a large increase of excess LOS, special attention should be paid to these.

## Abbreviations

AE: Adverse event
LOS: Length of stay
ICU: Intensive care unit
CI: Confidence interval
ICC: Intraclass Correlation Coefficient
